# Early research on anther‐smut disease: A fuller view of science?

**DOI:** 10.1002/ece3.11483

**Published:** 2024-05-30

**Authors:** Janis Antonovics, Helen M. Alexander

**Affiliations:** ^1^ Department of Biology University of Virginia Charlottesville Virginia USA; ^2^ Department of Ecology and Evolutionary Biology University of Kansas Lawrence Kansas USA

**Keywords:** human dimension in science, *Microbotryum lychnidis‐dioicae*, plant pathology, scientific method, *Silene latifolia*

## Abstract

The anther‐smut host–pathogen system has provided extensive insights into the evolutionary ecology of disease resistance, transmission modes, host shifts, pathogen specialization, and disease evolution in metapopulations. It also has led to unexpected insights into sex ratio distorters, sex chromosome evolution, and transposable elements in fungi. In addition, anther‐smut disease played a major role in Linnaeus' germ theory and the correspondence on parasitic castration between Darwin and Becker, one of the first female botanists. Here, we explicitly highlight some of the realities in the process of science, using an unusual autobiographical approach to describe how we came to collaborate on this system in the 1980s. Using perspectives from our different career stages, we present a surprising narrative that could not be deduced from merely reading the published papers. While our work was grounded in previous ecological and evolutionary theory, it was the product as much of empirical failures and intellectual roadblocks, as the result of a progressive scientific method. Our experiences illustrate not only the “human dimension of science” but more importantly show that linear sequences of hypothesis testing do not necessarily lead to new study systems and new ideas. We suggest there is a need to re‐evaluate the scientific method in ecology and evolution, especially where the challenge is to engage in a productive dialog between natural history and theory.

## Graphical abstract

This paper shows by example that developing new research directions in ecology and evolution is not an impersonal process of sequential hypothesis testing but necessarily includes a strong human dimension where timelines and diversity of people and places play major roles. These aspects cannot be deduced from the published literature. This account will be useful for teachers and aspiring scientists and challenge standard perceptions about how science operates in reality.

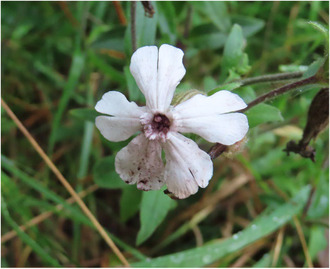

## 1 INTRODUCTION

The well‐established layout and style of scientific papers is designed to explain research clearly and objectively. However, no matter how truthful and honest a scientific paper is, it always deliberately obscures the humanity behind a study. The circumstances and human relationships that form the backdrop of any serious research, including the stress, the pain, and the joys, remain hidden in the published work. This disconnect between what we write in science and how it happens has been a major motivation for this account, which was stimulated in part by the difficulty one of us (JA) had when helping write an obituary for Jim Murray (1930–2023), former director of Mountain Lake Biological Station, the University of Virginia's field station. It was especially challenging trying to recreate the excitement and motivations that led to his classical work with Bryan Clarke (1932–2014) on speciation in the land snail *Partula* on the Polynesian Island of Moorea (Clarke & Murray, 1969), even though books describing this work are available (Gerlach, 2014, 2016). Remarkably little of the human background to research can be deduced by reading only published scientific papers, and biographies require drastic revision when notebooks and private correspondence become publicly available, as in the case of Louis Pasteur (Geison, 1995).

Another important concern is how to stimulate students contemplating a career in the sciences. Once in that career, reading papers is part of the necessary training of graduate students. Yet, for the undergraduate, reading what are often bland accounts of testing hypotheses that seem to come out of specialized concerns is unlikely to be inspirational. The reality is that scientific papers leave out so much that is interesting, and do not describe the emotions, nor the interaction of circumstance with personal and intellectual motivation that lead to successful research. So often we know nothing of the enjoyment or the community of nonscientists that are essential adjuncts to the process.

**FIGURE A1:**
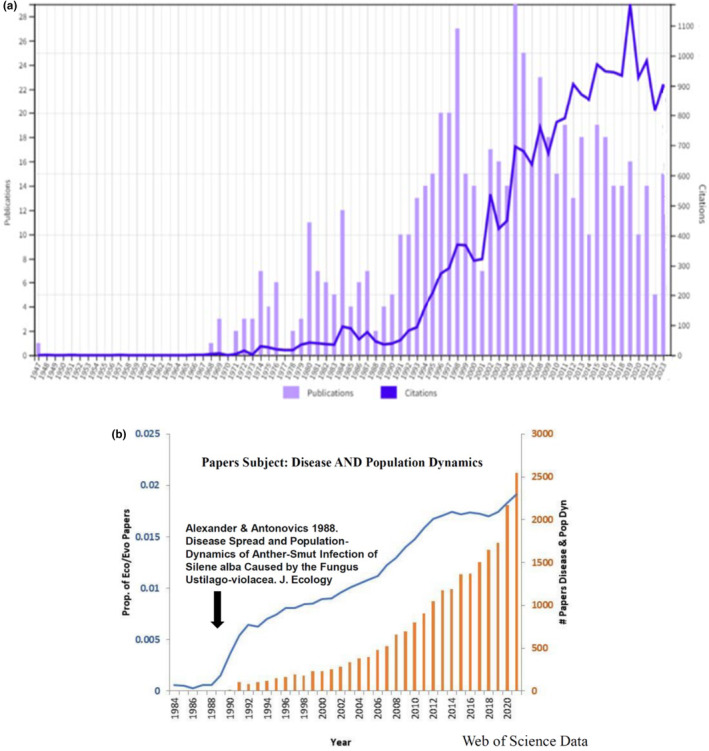
(a) Publication record for *Microbotryum*/*Ustilago violacea* from the Web of Science. Summary statistics: publications 627; times cited 20,972; *H*‐index 70 (https://www.webofscience.com/wos/woscc/basic‐search; accessed 5th February 2024). (b) Histogram (yellow bars) showing number of papers on disease and population dynamics. Solid line shows the proportion of papers on disease and population dynamics as a proportion of the number of papers on ecology and evolution during this period. The arrow shows the timing of the first joint publication by Alexander and Antonovics on anther‐smut disease dynamics. Provided by Michael Hood, Amherst College, 20th February 2024.

In this account, we explore the process of science, and its human dimensions, as it relates to the beginnings of our own collaborative research with anther‐smut disease. This seemingly obscure plant disease has become a well‐established model system for understanding infectious disease in natural populations (Appendix 1: Figure A1) and has thrown remarkable light on sexually transmitted diseases (Lockhart et al., 1996), on the ecology and evolution of metapopulations (Alexander et al., 2012; Hanski, 2001), and on the evolutionary genomics of sex chromosomes (Hood et al., 2004) and other areas of ecology and evolution (Appendix 2). We by no means initiated all this research but our initial collaboration (Appendix 3) provided a catalyst for some of these future studies.

**FIGURE 1:**
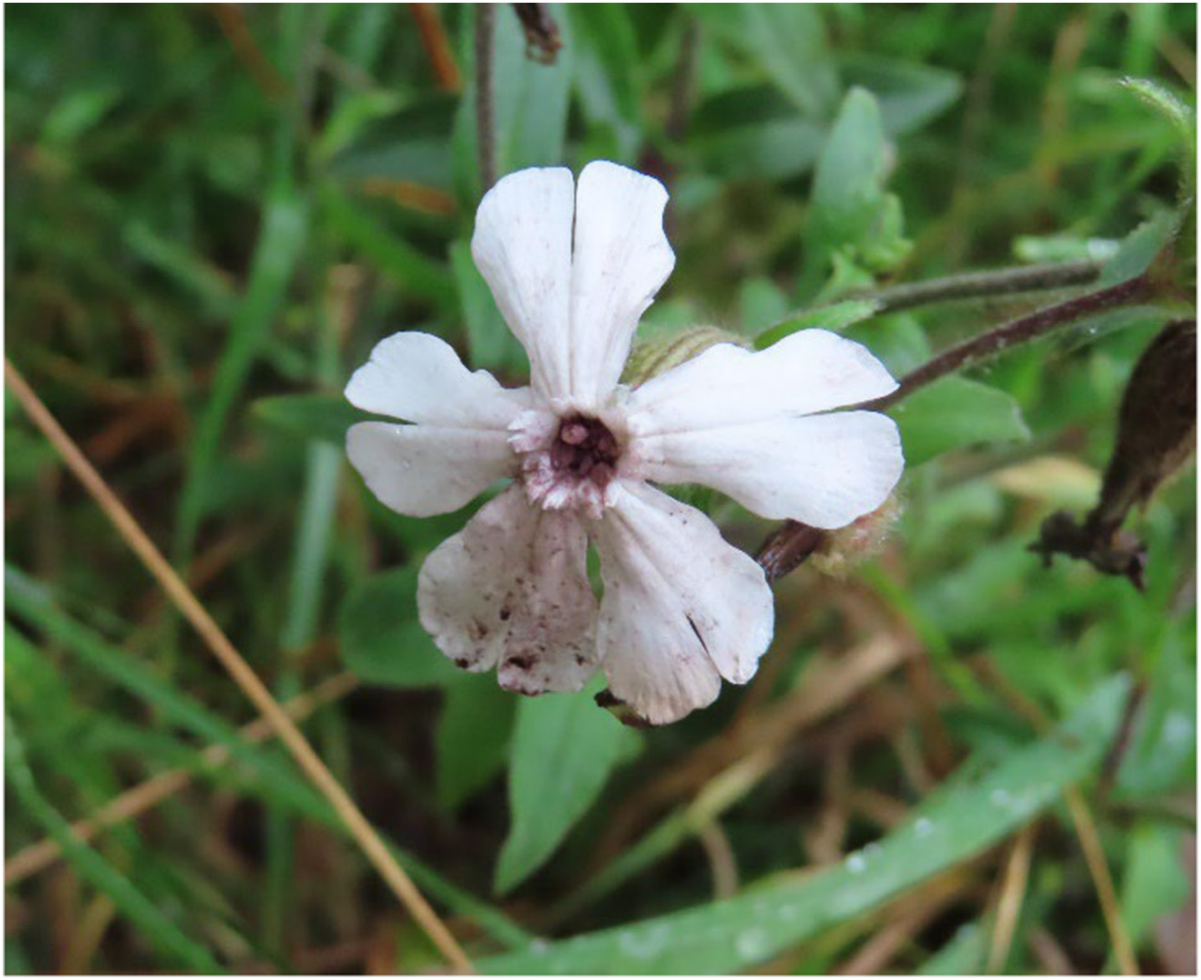
Flower of white campion, *Silene latifolia*, diseased with anther smut caused by *Microbotryum lychnidis‐dioicae*. The flower is of a female plant that has been forced by the fungus to develop stamens with anther sacs filled with dark‐purple spores that spill onto the petals. In males, the anthers would also be spore filled.

Anther‐smut diseases are caused by fungi that produce spores in the anthers of a flower and pollinators transmit these spores to new hosts. The best‐known anther smuts are in the genus *Microbotryum* and they infect plants in the carnation family (Caryophyllaceae). We studied the anther‐smut species (*Microbotryum lychnidis‐dioicae*) on white campion (*Silene latifolia*), a common roadside flower introduced into the United States about 200 years ago from Europe. The plants have separate male and female individuals, and the pathogen induces a morphological sex change in the females causing them to be male‐like by producing stamens with spore‐filled anthers and a reduced and sterile ovary (Figure 1).

Initially, we were unaware that anther‐smut disease had a famed academic pedigree; it had been studied by both Carl Linnaeus and Charles Darwin many years previously. For Linnaeus, it was a stimulus for proposing that microorganisms were a major cause of infectious disease not just in plants but also in humans; this was over 100 years before Pasteur's discoveries (Antonovics & Hood, 2018). Darwin was made aware of anther‐smut disease by Lydia Becker, better known for her contributions to women's suffrage (Gianquitto, 2013; Williams, 2022). In one of the first scientific papers by a female biologist, she describes the pathogen‐induced sex change and recounts her debate with Darwin about the cause (Becker, 1870). However, anther‐smut diseases became forgotten in the mainstream of ecology and evolution with the exception of Baker (1947) and the extensive genetic studies of Garber et al. (1975), reviewed in Garber and Ruddat (2002). Our “re‐discovery” of this system in the 1980s was therefore not the product of a rational progression from earlier studies but the indirect result of rescuing a failed dissertation project in a somewhat ornate and opportunistic manner that we believe is typical of many scientific discoveries.

Our paper has an unusual format. We separately describe the individual paths that led to our collaboration on the anther‐smut system, showing how our intellectual goals were intertwined with different personal circumstances. One of us at the time was a post‐doctoral researcher, but unpaid and looking for a permanent position. The other was a well‐established full professor at a major university but rather stuck on how to test a favored hypothesis and what to do next in research. We share our personal stories with two goals—illustrating the evolution of our ideas and hypotheses yet also revealing how happenstance, the people around us, and other unpredictable features influenced our work.

## 2 INDIVIDUAL PERSPECTIVES

### 2.1 Helen Alexander

*1977–1982 Graduate student; 1982–1983 Post‐doctoral Fellow, University of Minnesota; 1984–1987 Adjunct Assistant Professor and Research Associate, University of Louisville, Kentucky; 1988–1993 Assistant Professor, 1993–2003 Associate Professor, 2002–2023 Full Professor, 2023–present Professor Emerita, all at University of Kansas*.

I (Helen) started graduate school at Duke in 1977. I was fascinated by plant–animal interactions, a topic in vogue in the 1970s following the seminal work of Ehrlich and Raven (1964). I was also intrigued by examples of rapid evolution, such as Wu and Antonovics's (1976) work showing roadside plants had evolved lead tolerance (from lead in gasoline additives) in just decades. After several false starts on dissertation projects, I sought a local plant–herbivore system to study intraspecific variation in herbivory, which I saw as the first step in studying coevolution. I learned that a common butterfly (buckeyes, *Junonia coenia*) used ribwort plantains (*Plantago lanceolata*) as their larval host. This seemed like a perfect system but it was outside of my advisor's (Norm Christensen) expertise, so I switched labs, and Janis and Mark Rausher became my new co‐advisors. I knew Janis had studied *Plantago* while Mark had expertise in butterfly ecology. This switch was not only exciting but also a bit intimidating. Janis did not help by having skull and crossbones symbols on his door for times he did not want to be interrupted!

In 1979, I collected some baseline data on butterfly oviposition but the previously abundant buckeyes were suddenly rare that year. Panic set in as I no longer had a dissertation project. Luckily, Janis and I had noted a fungal disease on the inflorescences of *Plantago* that turned them leafy and likely reduced seed production. So without much thought, or even knowing what the fungus was, I switched my focus from plant–herbivore to plant–pathogen interactions. I relate these personal details to illustrate how much research failures (lack of the insect I wanted to observe) and curiosity (noticing a fungus when I was supposed to be looking for butterflies) led to my dissertation research. Since I knew nothing about fungi, I took courses in the Plant Pathology Department at nearby North Carolina State University, and Kurt Leonard, a population geneticist and pathologist, joined my graduate committee. When asked by plant pathologists what my interests were, I explained that I studied the ecological and evolutionary consequences of disease in natural as opposed to crop populations. This research area essentially did not exist at the time, with the important exception of studies of forest pathogens. When I attended meetings, my talks were placed in “plant‐herbivore” sessions since so few ecologists were interested in disease. However, my work coincided with several other scientists' early interests in disease in natural plant populations, notably Adrian Gibbs and Jeremy Burdon in Australia. When studying for prelim exams, I read John Harper's classic *Population Biology of Plants* and loved that he had a pathogen chapter.

With time, my research evolved. I became excited about testing hypotheses about the effects of infection on plant fitness and the factors (genetic and nongenetic) that determined intraspecific variation in infection. As an aside, despite many efforts, I failed to develop greenhouse inoculation protocols for my fungus. I instead devised field experiments that relied on natural infection processes, an approach that proved powerful and was important for my later anther‐smut work. I eventually learned the identity of the fungus as *Fusarium moniliforme* var. *subglutinans* (Alexander, 1984).

I wanted to continue studying plant disease after my Ph.D., so for my post‐doc, I joined the laboratories of Alan Roelfs and Jim Groth at the University of Minnesota and also collaborated with Jeremy Burdon who was a visiting scientist there. I was newly married to Dave Alexander (a former Duke graduate student) and he moved with me to Minnesota. To reciprocate, when Dave started teaching at Bellarmine College in late 1983, I moved to Louisville, Kentucky, and obtained an office at the University of Louisville. The only positions there were short‐term teaching jobs so I opted to focus on research (without a salary), hoping for a future faculty position. To make this work, I credit my husband for his flexibility and my parents who occasionally sent generous checks. I was actively looking for new projects and contacted Janis for advice.

Janis told me about seeing the anther‐smut fungus near Mountain Lake Biological Station. I was also interested in the possibility of summer funding and luckily the Station co‐directors (Jim Murray and Jim Riopel) had begun an initiative to promote research and were offering summer fellowships to young researchers. I developed a proposal on anther smut focusing on demography and factors affecting a plant's likelihood of infection. In the end, I received two summers of funding ($1000 each summer, plus room and board) and a Sigma Xi grant for $500. These were not huge amounts of money but extremely helpful to me at the time. I was delighted to find Herbert Baker's (1947) paper and I visited Ed Garber and Manfred Ruddat in Chicago to learn the basics of doing genetics with the fungus.

**FIGURE 2:**
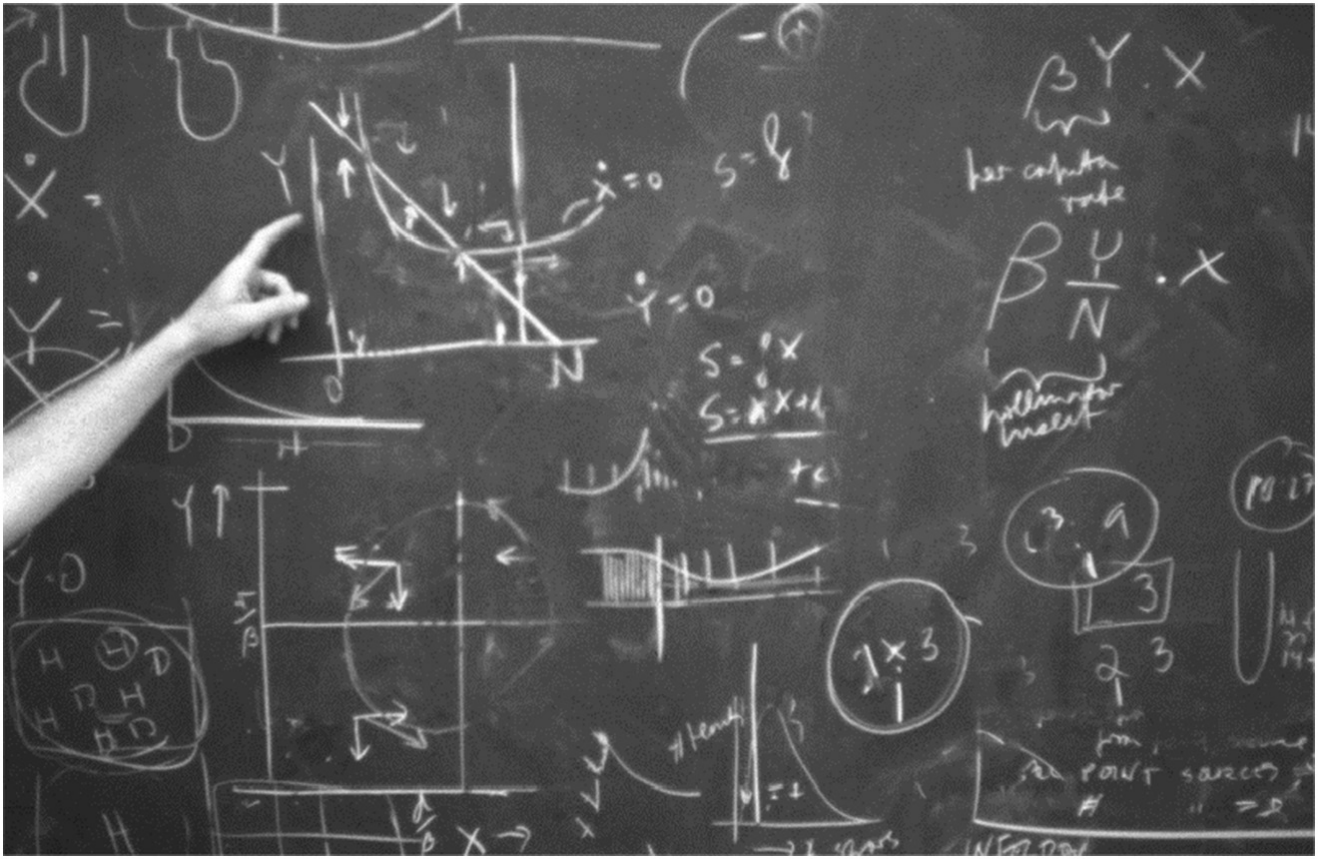
Brainstorming disease models on a blackboard at the Mountain Lake Biological Station in the late 1980s. The two alternate representations of transmission (βY and βY/N) can be seen in the top right.

My early work focused on a site at the Miles C. Horton Sr. Research Center, on a private estate owned by Ruth and Miles Horton who were strong supporters of the Biological Station. I started by permanently marking healthy and diseased plants for demographic studies (Alexander & Antonovics, 1988), and measured spore dispersal to gain insights on disease spread (Alexander, 1990a). Janis seemed intrigued by my findings and one evening he bombarded me with questions about the plant and fungus, and he started to parameterize population models. These exciting conversations (Figure 2) led to an early population dynamics paper (Alexander & Antonovics, 1988) and later experimental tests of frequency‐ versus density‐dependent disease transmission (Antonovics & Alexander, 1992). My first published *Silene* paper was, however, on pollination limitation (Alexander, 1987), a byproduct of other work when planned experiments failed (see below) and it was a paper that I could finish quickly.

I submitted NSF grants and was successful on my second try. I appreciated that the University of Louisville allowed me to submit the grant as a PI despite not having a faculty position (not all universities, including Duke, were willing to do this). Janis joined me as a co‐PI on my second NSF grant (1989–1991). Our goal was to explore the extent of genetic variation among plants in infection and variation in virulence in the fungus. Most pathologists study resistance variation by controlled inoculations where each plant receives the same dose of pathogen spores. Yet, in field situations, plants may have traits that increase the number of spores they encounter. My plan was to test hypotheses about floral traits, exploring whether genetically based variation in flower phenology or flower number altered insect visitation and therefore the probability or number of smut spores reaching the plant. To explore these ideas, we needed to measure resistance variation in a field‐based experimental plant population of known genetics, incorporating inoculated plants as a source of infection, and letting insects spread spores. It took me 3 years to establish this experimental population: competition from other plants, deer, and voles foiled my first two attempts. In retrospect, I wonder why I persevered! Janis' encouragement was essential—I recall him literally painting a picture in my mind about how a future successful study would help answer our questions. We also saw ways to improve our methodology, with more effective fencing and putting plants in sunken pots.

By my third attempt, we also had a better experimental plan. In systemic diseases like anther smut, individuals can only be scored as diseased or healthy. So the best option to assess disease susceptibility of an individual genotype was to measure the proportion of diseased plants in many replicates of cloned genotypes. Fortunately, Janis had free access to the phytotron facilities at Duke, a sophisticated plant growth facility. Janis volunteered to grow, clone, and inoculate my plants in Durham, and he was a huge help in establishing an experimental plant population with ~1000 plants.

Studies of this population proved fruitful. We documented extensive variation among plant genotypes in likelihood of infection, and male genotypes with more flowers and with earlier flowering were more susceptible (Alexander, 1989). We later created a similar field experimental population with progeny of crosses of these plant genotypes that confirmed the heritability of resistance variation and explored phenotypic and genetic correlations between disease and floral traits (Alexander & Antonovics, 1995). Our early studies also revealed that nonflowering plants could become infected, a result that encouraged us to examine this mode of infection in more detail (Alexander, 1990a; Roche et al., 1995).

Summers in Virginia were busy but it was not all work. My husband and I took many short trips in the area. He receives credit for discovering anther‐smut disease on the native fire pink, *Silene virginica*, when he noticed an odd‐looking flower while on a hike. This in turn led to future studies on the host range and specificity of *Microbotryum* (Antonovics et al., 1996). Although Dave was well established at Bellarmine College, he encouraged me to go on the job market, leading to positions for both of us at the University of Kansas. I negotiated my job details on a pay phone during a thunderstorm at the Station; emails were in their infancy and cell phones were far in the future.

The research I outlined above was done at or near the Station, but from early on I wanted to understand the broader dynamics of the disease. Was the disease common or rare in the surrounding mountains and valleys, and did it spread quickly or slowly? I also needed areas where the disease was absent to carry out studies of spore dispersal distances. For this work, I put diseased inflorescences in a container for 24 h and counted spores on healthy flowers at different distances from the point source (Alexander, 1990a). In the most isolated site, near the township of Maggie, several naturally occurring plants became unintentionally diseased, and there was an ensuing spread of the disease into nearby sections of roadside. How these experiments affected the regional dynamics of the disease in that valley is not known. Today I look back and wonder whether introducing this disease to new areas was appropriate. However, at a conference, John Harper enthusiastically praised my experimental approach of “releasing disease.” It was not without benefit, as the area of the unwanted diseased plant is the only place where we have long‐term dynamics in a single, well‐isolated area.

To examine overall disease trends across the county, we carried out surveys of plant populations in areas outside of the Station in 1984, and again in 1988. These studies showed that there was substantial turnover of both diseased and healthy populations (Alexander, 1990b). Janis then proposed extending the study by focusing on a larger area and doing it more systematically. This roadside survey approach made sense because *Silene* (and its pathogen) existed in mostly linear populations along disturbed roadside habitats. Working in one spatial dimension instead of two had great advantages for future analyses and theory.

I recall Janis describing the idea of the larger census to me with great enthusiasm, but then belatedly mentioning that he could not help since he was spending that summer in England. I forged ahead with my students and François Felber, a visiting researcher from Switzerland. Janis' idea was to count numbers of healthy and diseased plants in 44 yd (40 m) units. This strange measure is a quarter of a tenth of a mile and was chosen because it was measurable (at least approximately) on a car odometer (no GPS in the 1980s). We used local landmarks and prominent trees to pinpoint exact unit boundaries, admittedly with some confusion. For example, in field notes, Janis had recorded that lime trees grew in Virginia, but it had to be explained that for those like Janis with an English upbringing, “lime tree” was the common name for basswood or *Tilia americana*.

Doing the survey that first year was challenging: plant populations were not always strictly linear, landowners were curious about what we were up to, and we had to be careful with traffic. On Janis' return from England, the lab group, always eager to tease, labeled Janis “The Queen” (making honorary visits) while I was dubbed “The Prime Minister” (charged with getting things done). These early studies helped us initiate a long‐term study based on a larger spatial and metapopulation perspective (Antonovics, 2004), one that has continued into its 36th year.

During this period, Janis was getting more and more interested in disease and he asked me if he could submit his own NSF proposal. Janis recalls this as a sheepish request, but I was amazed that such an established scientist would so respectfully ask my opinion.

By this time, I was beginning my Kansas career and was a young mother. Summers at Mountain Lake Biological Station had been busy with research plus pregnancy, then a baby, and then a toddler. With a second child in 1991, I reluctantly decided that Virginia field research was too logistically challenging to keep up. Although I continued to collaborate with Janis and others on anther‐smut research, staying in Kansas was simpler for our family and I was developing new research projects in this prairie region (Alexander et al., 1997; Alexander & Mihail, 2000).

### 2.2 Janis Antonovics

*1963‐1966 Graduate student; Botany Department, Duke University: Professor 1977–1987; Director, University Program In Genetics 1984–1988; James J. Wolfe Professor, 1987–1998. Biology Department, University of Virginia: 1998–2015 Lewis and Clark Professor; 2015–present Research Professor*.

I (Janis) had spent the summer of 1979 in Bangor, North Wales, as a Visiting Professorial Fellow, and while there I had been shown anther‐smut disease on red campion by Glen Matlack, then a graduate student with John Harper. Glen was studying floral biology of members of the carnation family (Matlack, 1987; Matlack & Harper, 1987). In the summer of 1981, while driving up to Mountain Lake Biological Station to teach a summer course, I noticed white campion along the roadside. When I stopped at the pull‐off with a spectacular view of the New River Valley, I was surprised and delighted to find that some of the flowers had dark purple‐brown anthers unmistakable as anther‐smut infection. I could now show my summer class this “gee whiz” example of pathogen‐induced sex change within easy reach of the Station. However, I did not envisage it in any research context. Indeed, before Helen started with anther smut, I had little direct interest in plant diseases.

My main research topic at the time was carrying out field experiments to assess the relative success of sexual and asexual progeny, using the easily cloned sweet‐vernal grass *Anthoxanthum odoratum*. The experiments were in the same field in which Helen did her Ph.D. research. We showed that the advantages to genetically variable progeny were large and clear (Kelly et al., 1988), and genetic variation for resistance to pathogens or insect attack was implicated (Schmitt & Antonovics, 1986). However, I was soon at an impasse because identifying the pathogen or pathogens responsible seemed impossible; DNA methods for analyzing community composition of microorganisms had not even been dreamt of. I did have the idea of generating genetically uniform seed progeny using doubled haploid progeny from anther culture and then following their fate in the field (Kasperbauer & Eizenga, 1985), but in 6 months of considerable effort and time, I failed to regenerate green plants (plenty of roots though) from the callus tissue and had no ready solutions when the “standard” tissue culture recipes did not work. So I was very much stuck in terms of future directions.

My interactions with Helen at Mountain Lake Biological Station worked wonders to extract me from this intellectual and technical impasse. More than that, it resulted in a confluence of ideas that had been brewing in my head for a while, and which I thought were central in biology. I had become very interested in transmission as a general phenomenon common to genes, diseases, and culture. I invited Robert Boyd, co‐author of “Culture and the Evolutionary Process” (Boyd & Richerson, 1985), for a seminar at Duke. I was intrigued by Eshel's (1985) work on the maintenance of Mendelian segregation (a form of “controlled” or shared transmission) in the face of distorter genes and his explanation for the remarkable fact that there are almost no Eukaryotes that have only a single pair of chromosomes. I had also become interested in the evolution of uniparental inheritance, stimulated by Nick Gillham and John Boynton whose lab was opposite mine and with whom I taught Principles of Genetics. Their research was on *Chlamydomonas* (Boynton et al., 1987), a unicellular alga where curiously the chloroplast genome is inherited through the plus mating type, whereas the mitochondrial genome is inherited through the minus mating type. Uniparental inheritance was clearly not a simple consequence of gamete size differences in this isogamous species. My theoretical models showed that uniparental inheritance could have evolved as a mechanism preventing the spread of over‐replicative “selfish” organelles. Unfortunately, for my ego, I was scooped on this idea (Hurst & Hamilton, 1992; Law & Hutson, 1992) and it was another impasse as my results were not unique enough to publish.

It was the height of the AIDS epidemic, and while my personal fears were minimal, my interest was piqued by a seminar on the Ty repetitive sequences in yeast. The speaker (I cannot remember who) wondered what advantages such “junk” DNA could have, but it immediately seemed to me likely that such transposable elements could be transmitted following mating between strains that had them and those that did not. They were simply sexually transmitted diseases, proliferating in the genome. From an academic viewpoint, the idea that mating could simultaneously lead to genes and pathogens being transmitted seemed rich with possibility, an idea we later followed up on (Thrall et al., 1997).

At Mountain Lake Biological Station, I developed theoretical models of anther‐smut transmission with Helen (Figure 2). Not having access to the literature (no internet then), I assumed, largely based on the work of Don Levin, that pollinators adjusted their flight distances to compensate for host density (Levin & Kerster, 1974). I therefore made disease transmission a function of disease frequency (the probability that a pollinator would visit a diseased plant) rather than of the density of diseased plants. It soon became evident that frequency‐dependent disease transmission could result in population extinction, and that anther‐smut disease might have serious consequences for its host. I wrote to several people regarding the use of frequency‐dependent transmission for modeling disease, including Bob May who referenced me to the now seminal paper of Getz and Pickering (1983), and (true to form) to his own early mention of frequency‐dependent transmission in sexually transmitted diseases (May & Anderson, 1979). We subsequently set up experiments to show that frequency‐dependent transmission was likely important in the spread of anther smut (Antonovics & Alexander, 1992).

Given that anther smut might result in population extinction, how could it co‐exist with its host? Caswell (1978) showed that long‐term co‐existence was possible if unstable systems were spatially interconnected, and Levins (1969) introduced the concept of the metapopulation, a system of interconnected populations where, even with extinction of individual populations, colonization could stabilize the system. Ilkka Hanski was the first to instantiate Levin's ideas in a real‐world system, and I was stimulated by his work when he visited Duke. It was a particular delight when later at a conference he pointed out the remarkable similarities between “his” metapopulation of the Glanville fritillary butterfly (*Melitaea cinxia*) on ribwort plantain *P. lanceolata* off the coast of Sweden, and “our” metapopulation of anther‐smut and *Silene* in Virginia (Hanski, 2001). My own entry into these spatial aspects was very tentative, but greatly stimulated by conversations at Mountain Lake Biological Station with another young researcher, Dave McCauley. He, like Helen, was a Summer Research Fellow at the Station, and his work had shown that frequent colonization and extinction events greatly influenced genetic differentiation (Wade & McCauley, 1988). I remember asking Dave one evening at the Station, no doubt over a beer or two, whether it was worth continuing the annual census that we had started, and I was buoyed by his enthusiasm. Eventually, he himself would get involved in the study of “genetic rescue” using our historical data on the *Silene* populations around Mountain Lake (McCauley et al., 1995).

These disparate intellectual threads of the evolution of mating systems, ecological coexistence, and sexual transmission came together as a rich and tempting stew. My interactions with Helen at Mountain Lake therefore not only had a welcome freshness and novelty but resonated with many of my earlier interests. There was now so much to do and so many questions to ask. I was delighted that Helen was pleased for me to put in an independent NSF proposal side by side with her already well‐established studies. Anther smut had only been on my radar as a rather odd example of pathogen‐induced sex change, but in a year or two it had converted me to a “born‐again disease biologist” with almost an evangelical zeal and commitment to pursuing this quirky disease, and its implication for not just plant ecology and evolution but also animal (Antonovics & Edwards, 2011) and human diseases (Baker & Antonovics, 2012; Lockhart et al., 1996).

For me, the pursuit of anther‐smut disease, both literally and metaphorically, has continued to the present day, with wonderful students, collaborators, and colleagues too numerous to acknowledge individually (Appendix 2). Particular excitements have been the discovery of morphologically differentiated haploid sex chromosomes in the fungus, exploring sexually transmitted diseases in primates, and examining how disease might affect host species distributions. The enjoyments have been participating in field experiments and long‐term censuses both at Mountain Lake Biological Station as well as in the Italian Alps, a center of diversity for many species of anther smut and their hosts.

I have continued to interact with Helen in terms of ideas and approaches, becoming also involved with her sunflower research (Alexander et al., 2012; Moody‐Weis et al., 2008). For teaching, it was Helen who encouraged me to use student posters in my Biology of Infectious Disease course, an approach that students thoroughly enjoyed and which became infamous in the department for their graphic impact. The stimulus for the present paper was also thinking back to the beginnings of our collaboration while analyzing the long‐term census data on anther‐smut disease which we started 36 years ago.

## 3 CONCLUSION

Our involvement in anther smut was not a pre‐determined research program, but a nonlinear, human interaction dependent, and contingent journey only tangentially related to the science itself. We adopted the anther‐smut system more as a consequence of casual natural history and personal circumstance than by any more grandiose goal of advancing understanding of infectious diseases or plant pathology using an “ideal model system.” A fascinating aspect of biological evolution is that the shorter the time scale over which it is studied, the greater the amount of evolutionary change that is detected (Gingerich, 1983); over geologic periods, the changes seem directional and even “orthogenetic,” whereas over short so‐called micro‐evolutionary timescales, the changes are rapid, now forward now reversed, or in some other direction. Perhaps this is also true of the scientific enterprise; it may seem directional and even rational over a long period of time or in retrospect, yet at the local level it can be fluid, tortuous, and often takes unpredictable paths. Certainly, this account of how and why we ended up working on anther smut simply cannot be deduced from our papers from this period (Appendix 3). The sequence of ideas does not follow the publication dates of the papers, and the rational justifications for using a system, useful in paper introductions, do not reveal the circumstances and feelings that led to our engagement with it. Peter Medawar (1967) famously described science as “the art of the soluble,” but in our experience, it is, like many activities in life, the “art of enthusiasm and persistence.” For evolutionary biology, it is also the art of matching ideas to systems where they can be tested (Travis, 2006).

Studies by others, such as Garber and Ruddat on the genetics of *Microbotryum* or Hanski on metapopulations of butterflies, were not just informative but also stimulating and inspirational. Like surfers who rely on waves, science relies on prior knowledge to not only move forward but also to generate enthusiasm. The specific path that ensues is a mixture of chance, training, and local circumstances. The nice thing about science is that results, if exciting or useful, keep the waves going. Family and friends make pivotal contributions by supporting what to them must seem ill‐framed directions and tentative goals.

As we look back, we realize that the unpredictable challenges in our early work coexisted with a more traditional framework of science methodology. As an example briefly noted above, observations of insects visiting flowers inspired Janis to include frequency‐dependent disease transmission in early models. We then performed field experiments to test this assumption of transmission mode, and that led to more models. This blending of observation, experiment, and theory has been a mainstay of our work and those of our colleagues (Thrall & Jarosz, 1994; Uricchio et al., 2023). Our main point here is that only describing the intellectual context of our work misses other essential ingredients of the scientific enterprise (such as enthusiasm, persistence, and perseverance, interest in natural history, diversity of past experiences, and ability to collaborate). We find people with these qualities are the most successful (and enjoyable colleagues) at research institutions and indeed it is evidence of these traits that we look for in job interviews for faculty positions.

We hope this paper will be of use to young scientists embarking on a career by showing them that science is not just progressive hypothesis testing, but a much broader humanistic exercise. To us, research is an odd and exciting enterprise willed by enthusiasms for ideas and systems, an enterprise driven (but not directed) as much by emotion as by premeditated reasoning, and also rife with hesitations and uncertainties that in turn lead to new opportunities. We suspect the twists and turns described here are the rule rather than the exception, especially for research in ecology and evolution. So often a major challenge in these fields is matching tractable real‐world systems to new ideas, and in such situations, natural history observations, an openness to “trying out” new systems, and doing so proactively (as well as in desperation) seem part of the scientific method. It necessarily includes a strong human dimension where diversity of timelines, people, and places (and perhaps field stations; Michener et al., 2009) stand out as prime contributory factors.

Adventures with anther smut were intertwined with our curiosity about the natural world. Our discovery of infected *S. latifolia* and *S. virginica* (and other species as well) was, for example, initially a simple result of people enjoying being outside and looking closely at plants that interested them. So often we as scientists get caught up in the rush of taking data that we forget that being curious and open‐minded are essential traits for any researcher. For students, it is important to realize that unexpected observations may open up new avenues of research questions and approaches. Similarly, those of us who are mentors need to encourage students to take the time to explore natural settings and follow research paths that are out of the ordinary. Curiosity is often what first draws people to ecological and evolutionary fields—we must actively promote this approach to biology because overworked scientists can forget the power of looking at the world with truly open eyes.

Throughout this essay, we have deliberately avoided the word “serendipity” because it is a tautological construct contingent on ex post facto interpretation of success (Copeland, 2019). However, the unpredictability of the course of the events in our research is surprising, even to us looking backward. The challenge for sociologists and philosophers of science is that there still seem to be missing pieces to the puzzle of the scientific method, especially in ecology and evolution where the primary challenge is to engage in an effective dialectic between natural history and theory, and where hypothesis testing seems a subcomponent of a broader more inclusive methodology.

## AUTHOR CONTRIBUTIONS

**Janis Antonovics:** Conceptualization (equal); investigation (equal); methodology (equal); writing – original draft (equal); writing – review and editing (equal). **Helen M. Alexander:** Conceptualization (equal); investigation (equal); methodology (equal); visualization (equal); writing – original draft (equal); writing – review and editing (equal).

## CONFLICT OF INTEREST STATEMENT

None.

## Data Availability

There are no data files involved in the paper.

## APPENDIX 2

Examples of research topics explored with anther‐smut disease or the *Silene* metapopulation at Mountain Lake Biological Station. Only a single reference under each topic is included, and chosen to emphasize the contributions of investigators explicitly not mentioned in the text.

**Table:**

Topic	Representative publications
Host–pathogen dynamics	Thrall, P. H., & Jarosz, A. M. (1994). Host‐pathogen dynamics in experimental populations of *Silene alba* and *Ustilago violacea*. I. Ecological and genetic determinants of disease spread. *Journal of Ecology*, 82, 549–559.
Metapopulation dynamics	Tellier, A., Villaréal, L. M. M. A., & Giraud, T. (2005). Maintenance of sex‐linked deleterious alleles by selfing and group selection in metapopulations of the phytopathogenic fungus *Microbotryum violaceum. American Naturalist*, 165, 577–589.
Sexually transmitted diseases	Wennstrom, A., & Ericson, L. (2003). The concept of sexually transmitted diseases in plants: Definition and applicability. *Oikos*, 100, 397–402.
Vector transmission	Real, L. A., Marschall, E. A., & Roche, B. M. (1992). Individual behavior and pollination ecology: Implications for the spread of sexually transmitted plant diseases. In D. L. DeAngelis & L. J. Gross (Eds.), *Individual based models and approaches in ecology* (pp. 492–508). Chapman and Hall/CRC.
Host–pathogen coevolution	Kaltz, O., Gandon, S., Michalakis, Y., & Shykoff, J. A. (1999). Local maladaptation in the anther‐smut fungus *Microbotryum violaceum* to its host plant *Silene latifolia*: Evidence from a cross‐inoculation experiment. *Evolution*, 53, 395–407.
Speciation	Giraud, T., Refrégier, G., Le Gac, M., de Vienne, D. M., & Hood, M. E. (2008). Speciation in fungi. *Fungal Genetics and Biology*, 45, 6791–802.
Host shifts and pathogen evolution	De Vienne, D. M., Hood, M. E., & Giraud, T. (2009). Phylogenetic determinants of potential host shifts in fungal pathogens. *Journal of Evolutionary Biology*, 22, 2532–2541.
Host life history variation	Bruns, E., Hood, M. E., & Antonovics, J. (2014). Rate of resistance evolution and polymorphism in long‐ and short‐lived hosts. *Evolution*, 69, 551–560.
Fungal mating type biases	Thomas, A., Shykoff, J., Jonot, O., & Giraud, T. (2003). Sex‐ratio bias in populations of the phytopathogenic fungus *Microbotryum violaceum* from several host species. *International Journal of Plant Sciences*, 164, 641–647.
Sex‐chromosome evolution	Bazzicalupo, A. L., Carpentier, F., Otto, S. P., & Giraud, T. (2019). Little evidence of antagonistic selection in the evolutionary strata of fungal mating‐type chromosomes (*Microbotryum lychnidis‐dioicae*). *G3 Genes|Genomes|Genetics*, 9, 1987–1998.
Sex‐ratio distorters	Taylor, D. R. (1999). Genetics of sex ratio variation among natural populations of a dioecious plant. *Evolution*, 53, 55–62.
Transposable elements	Horns, F., Petit, E., & Hood, M. E. (2017). Massive expansion of *Gypsy*‐like retrotransposons in *Microbotryum* fungi. *Genome Biology and Evolution*, 9, 363–371.
Genetic rescue	Richards, C. M., Emery, S. N., & McCauley, D. E. (2003). Genetic and demographic dynamics of small populations of *Silene latifolia. Heredity*, 90, 181–186.
Genetic structure	Keller, S. R., & Taylor, D. R. (2010). Genomic admixture increases fitness during a biological invasion. *Journal of Evolutionary Biology*, 23, 1720–1731.
Evolution of gynodioecy	Miller, I., & Bruns, E. (2016). The effect of disease on the evolution of females and the genetic basis of sex in populations with cytoplasmic male sterility. *Proceedings of the Royal Society of London, Series B: Biological Sciences*, 283, 20153035.
Fungal karyotypes	Perlin, M. H. (1996). Pathovars or *formae speciales* of *Microbotryum violaceum* differ in electrophoretic karyotype. *International Journal of Plant Sciences*, 157, 447–452.
Sexual conflict	Delph, L. F., Brown, K. E., Ríos, L. D., & Kelly, J. K. (2022). Sex‐specific natural selection on SNPs in *Silene latifolia*. *Evolution Letters*, 6–4, 308–318.
Evolution in invasive species	Wolfe, L. M., Elzinga, J. A., & Biere, A. (2004). Increased susceptibility to enemies following introduction in the invasive plant *Silene latifolia*. *Ecology Letters*, 7, 813–820.

## APPENDIX 3

Publications by Alexander and Antonovics relating to anther‐smut disease covering the early years of their research 1987–1994 (in chronological order).
